# Modification of Y-incision technique with Valsalva graft to enlarge subvalvular space

**DOI:** 10.1016/j.xjtc.2026.102294

**Published:** 2026-02-26

**Authors:** Akihiro Higashino, Yoshitsugu Nakamura, Taisuke Nakayama, Yuto Yasumoto, Kusumi Niitsuma

**Affiliations:** a Department of Cardiovascular Surgery, Chiba-Nishi General Hospital, Matsudo, Japan; b Department of Cardiovascular Surgery, Mitsui Memorial Hospital, Tokyo, Japan

**Figure:**
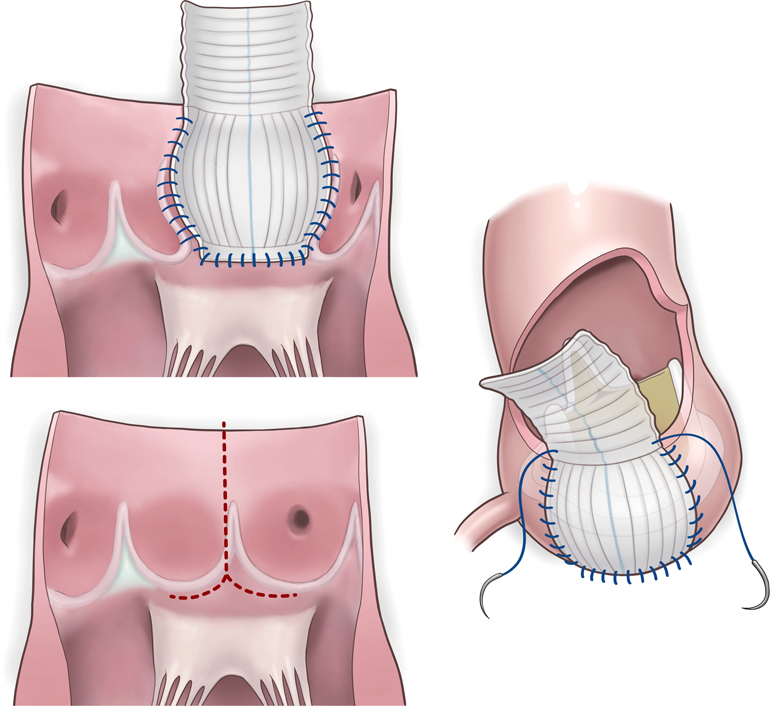
Valsalva graft patch allows transverse enlargement of the subvalvular space. @MEDICAL FIG

**Central Message**

A modified Y-incision with a Valsalva graft expands the subvalvular space, enabling larger valve implantation and potentially enhancing postoperative hemodynamics.

The Y-incision aortic annular enlargement technique, introduced by Dr Bo Yang in 2021, has been shown to effectively enlarge small aortic annuli, allowing for implantation of larger prosthetic valves while preserving structural integrity.^1^ However, when implanting a larger prosthetic valve after annular enlargement, it is important to ensure that adequate space is provided below the valve. If the prosthetic valve is oversized for a restricted subvalvular space, it may not function optimally, potentially impairing hemodynamics and reducing the durability of the valve. We report a modification to the conventional Y-incision technique using a Valsalva graft instead of a Dacron patch to optimize subvalvular space and improve hemodynamic performance.

## Surgical Technique

After a standard Y-incision is made, a transverse aortotomy is performed, and the aortic valve cusps are excised. The incision is extended through the annulus between the noncoronary and left coronary cusps into the aortomitral curtain, parallel to the annulus, reaching the nadirs.

The annular enlargement width is measured, and the graft size is calculated: (width + 5 mm) × 3 ÷ 3.14. For practical application, this calculation can be simplified to an approximate trimming length of width +5 mm, which provides sufficient accuracy, given that the Valsalva graft is available in 2-mm size increments. One-third of the J-graft Valsalva graft's circumference is trimmed. The graft is sutured to the aortomitral curtain using running 4-0 PROLENE suture, with care taken to avoid contact with the anterior mitral leaflet. The suture should be placed at the junction between the skirt and Valsalva portions to prevent lateral expansion from interfering with the mitral valve.

**Figure 1:**
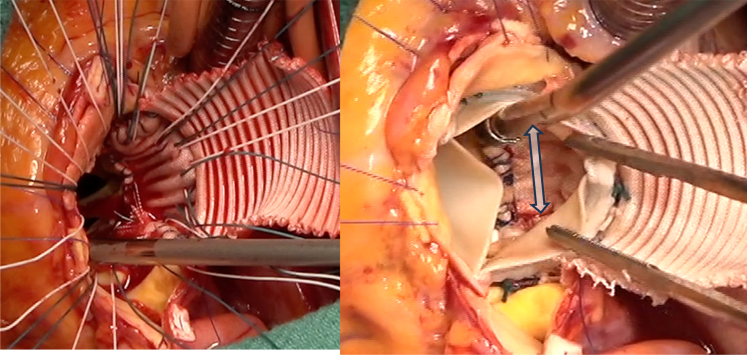
A Y-incision technique is applied, and the subvalvular space is enlarged using a Valsalva graft.

After reaching the fibrous trigones, the annular segments are closed up to the aortotomy. Noneverting mattress sutures with pledgets are placed along the native annulus. On the graft portion, nonpledgeted sutures are placed from outside to inside in an inverted U-shape to preserve the subvalvular space (Figure 1). Prosthesis orientation is confirmed using a sizer to avoid interference with coronary ostia, and the valve is implanted (Video 1).

This study was approved by the institutional review board of Chiba-Nishi General Hospital (approval no. TGE02861-025; approved on August 4, 2025). All patients provided informed consent for the publication of their clinical data in this study.

## Results

Between April 2023 and December 2024, we performed aortic valve replacement (AVR) using this modified technique in 8 patients, all of whom were female with a mean body surface area of 1.42 m^2^. In all cases, preoperative aortic annular measurements using computed tomography angiography predicted difficulty in implanting a 19-mm prosthetic valve. A 23-mm bovine pericardial bioprosthesis, equivalent to a 3-size increase, was successfully implanted in all patients (Table 1).

**Table 1:** Preoperative characteristics and operative data

Variables	Value (N = 8)
Preoperative characteristics	
Age, y	70.4 ± 7.1
Sex, male/female	0/8
Body surface area, m^2^	1.42 ± 0.16
Annulus diameter, mm	17.9 ± 1.6
Operative data	
Operation time, min	280.3 ± 52.3
Cardiopulmonary bypass time, min	165.5 ± 27.1
Aortic crossclamp time, min	118.8 ± 17.3
Prosthetic valve size, mm	23.3 ± 0.7
Valsalva graft size, mm	25.8 ± 2.0
Concomitant procedure, n (%)	4 (50%)
Operative mortality	0

Values are presented as mean ± standard deviation or number (%).

Postoperative echocardiography showed no significant paravalvular leak or new mitral regurgitation. Mean pressure gradient improved from 58.3 ± 15.2 mm Hg to 11.4 ± 5.1 mm Hg. Computed tomography confirmed that the subvalvular area increased from 265 ± 55 mm^2^ to 357 ± 40 mm^2^ (Table2). However, in 4 of 8 patients, the valve-to-coronary (VTC) distance for the right coronary artery (RCA) was ≤4 mm, raising concerns regarding future valve-in-valve (ViV) procedures.

**Table 2:** Pre- and postoperative data of echocardiography and computed tomography

Variables	Preoperative	Postoperative
Left ventricular dimensions		
LVDd, mm	41.1 ± 4.0	38.2 ± 1.8
LVDs, mm	23.5 ± 2.0	26.5 ± 2.2
LVEF, %	66.0 ± 4.3	59.0 ± 6.1
Mitral regurgitation (≥moderate)	0	0
Aortic valve		
Vmax, m/s	4.6 ± 0.5	2.2 ± 0.4
Mean pressure gradient, mm Hg	58.3 ± 15.2	11.4 ± 5.1
Area of subvalvular space, mm^2^	265 ± 55	357 ± 40
Valve-to-coronary distance		
LCA, mm	–	8.1 ± 1.4
RCA, mm	–	3.6 ± 1.3

Values are presented as mean ± standard deviation. *LVDd*, Left ventricular diastolic diameter; *LVDs*, left ventricular systolic diameter; *LVEF*, left ventricular ejection fraction; *Vmax*, maximum transvalvular velocity; *LCA*, left coronary artery; *RCA*, right coronary artery.

## Comment

With increasing transcatheter AVR and ViV use, ensuring future feasibility through implantation of larger prosthetic valves is crucial. The durability of prosthetic valves after AVR and the potential for future ViV procedures highlight the importance of implanting a larger valve. Consequently, the Y-incision technique introduced by Dr Bo Yang has gained widespread acceptance.

Unlike the Manouguian or Kanno techniques, which extend the incision into the left ventricular outflow tract (LVOT), the Nicks and Y-incision techniques primarily enlarge the annulus, and may not sufficiently expand the LVOT, while avoiding extension toward the anterior mitral leaflet and minimizing the risk of mitral valve interference. This can lead to the mismatch of an oversized bioprosthesis and a nonenlarged subvalvular space. Cleveland and colleagues^2^ reported that bioprosthetic oversizing impairs hemodynamic performance in vitro. Using an oversized prosthetic valve shifts the hinge point of the valve inward, negatively impacting hemodynamics.

**Figure 2:**
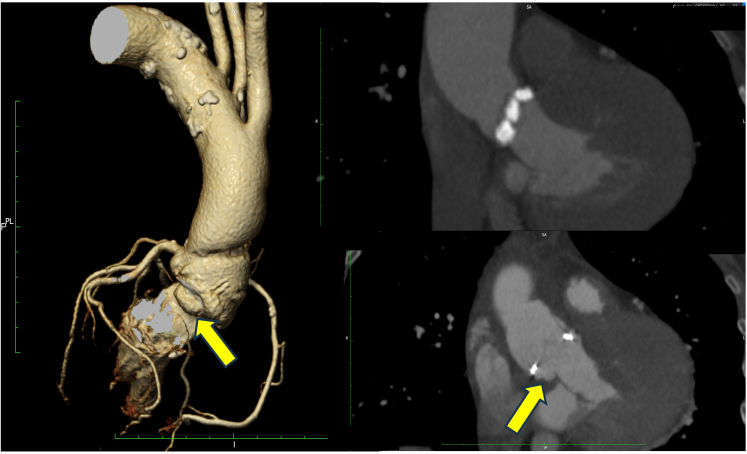
The *yellow arrow* indicates the bulging portion of the Valsalva graft beneath the prosthetic valve, which contributes to a smooth transition from the LVOT to the aortic valve. *LVOT*, Left ventricular outflow tract.

In the conventional Y-incision technique, the suturing of the prosthetic valve to the patch is performed in an inverted U-shaped fashion, which expand the space under the valve longitudinally. However, this may still result in a steep angle between the larger valve and the unexpanded LVOT. To address this issue, we used a Valsalva graft to expand the space under the valve in a transverse direction, promoting a smoother transition from the LVOT to the aortic valve (Figure 2). Although this technique does not directly enlarge the LVOT itself, it increases the subvalvular space, thereby potentially improving prosthetic valve performance. In cases where LVOT narrowing was considered critical, concomitant septal myectomy was performed. VTC distance is vital for ViV planning. Although all patients had adequate left coronary artery-VTC (≥4 mm), RCA-VTC was ≤4 mm in half of the cases. One possible explanation for this finding is that in AVR with annular enlargement using the Y-incision technique, the prosthetic valve tends to tilt toward the RCA. Furthermore, it has been reported that individuals of Asian descent, particularly women, tend to have relatively smaller diameters of the sinus of Valsalva and the sinotubular junction compared with other ethnic groups.^3^ This anatomical characteristic may partly explain the greater prevalence of cases with a smaller VTC distance observed in our study. Supporting this notion, data from Japan have demonstrated that the VTC distance may decrease after annular enlargement using the Y-incision technique,^3^ suggesting that this finding is related to the relationship between prosthesis size and a small aortic root, rather than the specific use of Valsalva graft. Adjunctive strategies such as sinotubular junction enlargement like Roof procedure,^4^ valve downsizing, or selection of a low profile prosthesis may help address this limitation. In conclusion, the modified Y-incision with a Valsalva graft offers improved subvalvular space and enables safe implantation of larger prostheses. Caution is warranted regarding RCA-VTC, and further refinements are needed to improve ViV feasibility.

### Declaration of Generative AI and AI-Assisted Technologies in the Writing Process

During preparation of this work the authors used ChatGPT in order to correct grammar mistakes. After using this tool/service, the authors reviewed and edited the content as needed and take full responsibility for the content of the publication.

## Conflict of Interest Statement

The authors reported no conflicts of interest.

The *Journal* policy requires editors and reviewers to disclose conflicts of interest and to decline handling or reviewing manuscripts for which they may have a conflict of interest. The editors and reviewers of this article have no conflicts of interest.

## Supplementary Data

Video 1 A Y-shaped aortic annular incision followed by sizing and trimming of a Valsalva graft used as a patch enables transverse enlargement of the subvalvular space. Video available at: https://www.jtcvs.org/article/S2666-2507(26)00101-X/fulltext.
