# Supplementary figures and images for: Modification of Y-incision technique with Valsalva graft to enlarge subvalvular space
Source: JTCVS Tech. 2026 Feb 26;37:102294. doi: 10.1016/j.xjtc.2026.102294 (PMC13261199; doi:10.1016/j.xjtc.2026.102294)

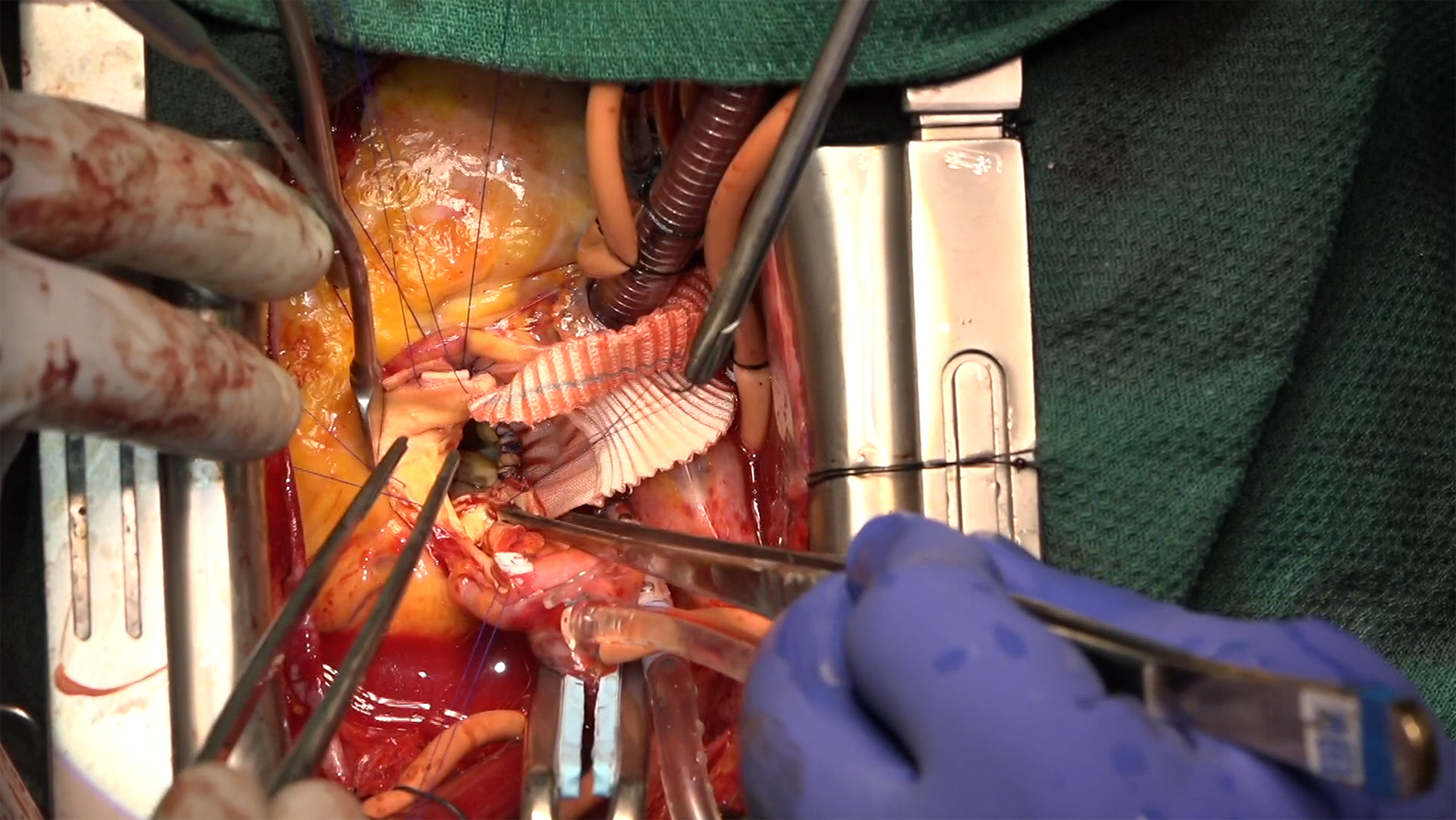

Supplement: Video 1 — A Y-shaped aortic annular incision followed by sizing and trimming of a Valsalva graft used as a patch enables transverse enlargement of the subvalvular space. Video available at: https://www.jtcvs.org/article/S2666-2507(26)00101-X/fulltext. [file fx2.jpg]
